# The Long Arm of Child Maltreatment and Mental Health in Later Life: The Effects of Maltreatment and Family Context?

**DOI:** 10.1093/geroni/igab046.1940

**Published:** 2021-12-17

**Authors:** Chengming Han, Tirth Bhatta, Eva Kahana, Brian Gran

**Affiliations:** 1 Case Western Reserve University, Cleveland, Ohio, United States; 2 University of Nevada, Las Vegas, Nevada, United States

## Abstract

Purpose. This article examines the role of family context in shaping the influence of childhood maltreatment on later life psychological well-being in the cultural context of Chinese society. Method. Data were drawn from the China Health and Retirement Longitudinal Study (CHARLS) baseline. Maltreatment was measured by corporal punishment by either mother or father in childhood. We used family violence, parents’ family socioeconomic status (SES) and mental health to represent family context. Result. Our ordinary least square regression analysis shows that corporal punishment administered by a mother was associated with higher depressive symptoms (b=0.308, p<0.05) in later life while being hit by father did not result in higher depressive symptoms. Family contexts had residual (“long arm”) influence on respondents’ mental health: violence in the family, including being hit by siblings (b=0.657, p<0.001) and witnessing violence between parents (b=0.658, p<0.001) contributed significantly to higher depressive symptoms. Conclusion. Corporal punishment by parents had long term effects on mental health of their children in later life. Cultural values, such as filial piety did not eliminate the negative impacts of being hit in childhood on mental health in later life. Family contexts including violence between parents also played important roles in shaping the relationship between child maltreatment and mental health in later life. Implication. Our study offers important insights about the complex matrix of cultural traditions, social circumstances and diversity in dealing with child rearing stress and their consequences for later life mental health.

